# A Study of Respiratory Hygiene Among Patients With Respiratory Manifestations in a Selected Primary Healthcare Setting in India

**DOI:** 10.7759/cureus.70864

**Published:** 2024-10-04

**Authors:** Arulprakash S, Nirmal Sujitha, Lovling Maria, Vijayakarthikeyan M, Minthami Sharon P

**Affiliations:** 1 Community Medicine, Vinayaka Mission's Kirupananda Variyar Medical College &amp; Hospitals, Vinayaka Mission's Research Foundation, Salem, IND; 2 Community Medicine, Vinayaka Mission's Kirupananda Variyar Medical College & Hospitals, Salem, IND; 3 Obstetrics and Gynaecology, Sree Balaji Medical College & Hospital, Chennai, IND

**Keywords:** oral hygiene practices, presumptive tb, pulmonary tuberculosis, respiratory hygiene, sputum disposal

## Abstract

Background: Tuberculosis is the second leading infectious killer disease, mainly transmitted through undiagnosed presumptive and inadequately treated pulmonary tuberculosis (PTB) cases in the community.

Objective: This study was conducted to assess the knowledge, attitude, and practices (KAP) related to respiratory hygiene and sputum disposal methods and to estimate the proportion of presumptive PTB cases among patients with clinical features of respiratory manifestations.

Methodology: A hospital-based cross-sectional study was conducted among 80 adult patients with respiratory manifestations at an urban health centre in Goa using a validated, pre-tested, semi-structured questionnaire by interview method.

Results: Among 80 study participants, 10 (12.5%) presumptive PTB cases were found. The knowledge regarding the exact cause and transmission of tuberculosis was 55% (44) and 72.4% (58), respectively. Around 32.5% (26) of participants were not following cough etiquette and they studied only up to middle school. Regarding the attitude towards ideal sputum disposal methods, more than three-fourths (81.3%, 65) of the responses were towards unsafe sputum disposal methods.

Conclusion: There is a need for health education regarding proper respiratory hygiene and sputum disposal methods, especially among patients with low educational and socio-economic groups.

## Introduction

Tuberculosis is the second leading infectious killer disease after COVID-19. The highest incidence of new cases is majorly recorded in low and middle-income group countries belonging to the African and South-East Asian regions where India tops in recording new cases [1]. An untreated sputum-positive pulmonary tuberculosis (PTB) case can infect 10-15 persons annually [1]. The main sources of tuberculosis infection are undiagnosed, diagnosed not adequately treated, and relapsed cases of PTB in the community, who are not practising proper respiratory etiquette, which predisposes to the transmission by droplet infection or droplet nuclei. Undiagnosed PTB or “presumptive PTB” case refers to a person with any symptoms and signs suggestive of PTB, including productive cough for more than two weeks, fever for more than two weeks, significant weight loss, haemoptysis, and any abnormalities in chest radiograph according to the Revised National Tuberculosis Control Programme (RNTCP) guidelines 2016 [2]. As per the India TB Report 2019, 6% to 10% of presumptive tuberculosis cases were confirmed for PTB [3]. Studies from other low and middle-income group countries reported that the prevalence of presumptive tuberculosis cases among the household contacts of PTB was around 7.5% and out of them, 17% of individuals did not seek any healthcare services, which further increases the TB disease burden in those countries [4,5]. Cough with expectoration, dry cough, runny nose, sneezing, sore throat, headache, and fever were the common respiratory manifestations that generate secretions or aerosols, and require proper respiratory hygiene to be practised. Safe sputum disposal method is recommended to prevent the further spread of tuberculosis by the Centre for Disease Control defined as pouring boiling water in the sputum container, spitting in a bactericidal container (containers with 5% phenol/cresol/sodium hypochlorite), or spitting on a paper and burning it [6]. But it was less practised where the majority follow unsafe sputum disposal by spitting indiscriminately in a public dustbin, gutter, washbasin, or dumping in the ground. Acquiring understanding and consistently practising proper cough and sneeze etiquette is a crucial preventive measure for managing infections in hospital environments. Even though information on cough etiquette is widely available, noncompliance in this regard makes it more difficult to prevent respiratory illnesses that spread through the air. The objectives of the study were to assess the knowledge, attitude, and practices (KAP) related to respiratory hygiene and sputum disposal methods among those patients who presented with respiratory manifestations and to estimate the proportion of presumptive PTB cases among the outpatients presenting with respiratory manifestations in a primary healthcare setting. Assessing the KAP related to respiratory hygiene and ideal sputum disposal methods among these patients will be helpful in spreading awareness, which may lead to a break in the transmission of PTB, which will eventually control the disease burden.

## Materials and methods

Study design

This was a hospital-based cross-sectional study that aimed to assess the KAP related to respiratory hygiene and sputum disposal among patients with respiratory manifestations in a primary healthcare setting. The study design was chosen because it allows for the observation of the target population at a specific point in time, helping to gather relevant data about respiratory hygiene practices within a limited timeframe.

Study setting

The study was conducted at the Urban Health Centre, Santa Cruz, which is located on the outskirts of Panaji, the capital city of Goa. This health centre operates under the direct administrative control of Goa Medical College and provides essential healthcare services, including outpatient care, basic emergency care, and maternal and child health (MCH) services. Additionally, special clinics are held weekly for the local population.

The medical staff at the centre includes five junior residents from the Department of Community Medicine, one medical officer, one in-charge doctor, and four interns. These healthcare workers ensure that the centre operates daily during the daytime, catering to a diverse patient population. This setting provides a representative sample of patients from both chronic and acute disease categories, making it an ideal location to study respiratory hygiene practices.

Study population

The Urban Health Centre caters to approximately 50 outpatients per day. About 80% of the patients attending the centre suffer from chronic illnesses such as diabetes, hypertension, and dyslipidemia. The remaining 20% consists of patients presenting with acute conditions such as fever, respiratory infections, diarrheal diseases, skin diseases, and other acute illnesses. The study population consisted of outpatients aged 18 years and above who presented with respiratory symptoms, including cough with expectoration, dry cough, runny nose, sneezing, sore throat, headache, and fever. These respiratory manifestations are important as they are commonly associated with conditions like tuberculosis (TB), which is highly prevalent in India.

Study period

The study was conducted over a period of two months, from December 2018 to January 2019. This limited time frame was sufficient to capture a sample of patients presenting with respiratory symptoms during this period.

Sampling technique

The period sampling method was employed to select participants. In this technique, all patients presenting to the Urban Health Centre, Santa Cruz, with respiratory manifestations during the study period were included as study participants. By the end of the study, a total of 80 patients were enrolled.

Study participants

The inclusion criteria for the study were outpatients aged 18 years and above, presenting with respiratory manifestations as listed earlier, and willing to provide informed consent. No exclusion criteria were mentioned, indicating that all eligible patients who visited the centre and presented with respiratory symptoms were included.

Ethics committee approval

Prior to the commencement of the study, approval (IHEC/GMC/2018/12) was obtained from the Institutional Ethics Committee of Goa Medical College. This ensured that the study was conducted in line with ethical guidelines, including respect for patient privacy, informed consent, and the protection of vulnerable populations.

Study tool

A validated, pre-tested, semi-structured questionnaire was used to collect data. The questionnaire was designed to gather information on the following: socio-demographic background of the study participants, clinically relevant manifestations for presumptive PTB as outlined by the National Tuberculosis Elimination Program (NTEP), knowledge about respiratory hygiene, cough etiquette, and appropriate sputum disposal methods, attitudes regarding the ideal respiratory hygiene and sputum disposal practices, and behavioural practices that could prevent the person-to-person spread of TB. The questionnaire was administered by trained personnel, ensuring consistency in data collection.

Data collection

Data collection followed these steps: informed written consent was obtained from each study participant, ensuring that they understood the study's purpose and agreed to participate. The questionnaire was administered through interviews with the patients, covering socio-demographic details, clinical history, and knowledge and practices concerning respiratory hygiene and sputum disposal. Participants were also asked about their attitudes towards safe respiratory hygiene practices and their behavioural practices related to cough etiquette and sputum disposal. At the end of each interview, healthcare workers provided educational guidance to the participants, emphasizing the importance of safe respiratory hygiene and proper sputum disposal methods. The goal was to improve patient practices to prevent the spread of PTB and other respiratory diseases.

Socio-economic classification

To assess the socio-economic status of the study participants, the modified BG Prasad Socio-Economic Scale (2019) was used. This classification system, introduced in 1961, is based on the per capita monthly income and can be applied to both urban and rural populations. It is updated regularly to account for inflation, making it a reliable tool for socio-economic assessment in contemporary studies [7].

Data entry and statistical analysis

The collected data were entered into SPSS version 22.0 (IBM Corp., Armonk, NY) for analysis. Descriptive statistics were used to summarize the data in terms of categorical variables as frequency and proportions. The chi-square or Fischer exact test was applied to assess associations between categorical variables, particularly regarding socio-economic status, educational background, and knowledge/practice of respiratory hygiene and sputum disposal. A p-value of less than 0.05 was considered statistically significant, indicating that any observed differences were unlikely to have occurred by chance.

## Results

The mean age of the study participants was 39 ± 15.97 years. The majority of the participants (65%, 52) were females in this study. During the study, the patients reported various respiratory-related manifestations, the most common being cough with expectoration, followed by dry cough, runny nose, sneezing, throat irritation, headache, fever, and difficulty in breathing. There were 10 presumptive PTB cases among the total of 80 study participants in the study. The proportion of presumptive PTB cases in this study was 12.5%. The obtained information was divided as “up to middle school” and “above middle school” for the education level variable and “middle class and below” and “above middle class” for the socioeconomic status variable. As per the educational level of the study participants, 45% (36) and 55% (44) of participants were from “up to middle school” and “above middle school” groups, respectively. As per the socio-economic status of the study participants, 67.5% (54) and 32.5% (26) of participants were from the “middle class and below” and “above middle class” categories, respectively. Table 1 describes the classification based on education.

**Table 1:** Knowledge and practices on respiratory hygiene & sputum disposal practices followed by the study participants according to the education level. * Multiple responses - total does not add to 100%. Fischer's exact test. A p-value of less than 0.05 is considered significant.

Knowledge and practices on respiratory hygiene & sputum disposal practices				
	Educational level			
	Up to middle school	Above middle school	Total	P-value
	n (%)	n (%)	n (%)	
Practices				
1) Do you cover your nose and mouth while coughing/sneezing/blowing your nose?				
Yes	10 (27.7)	44 (100.0)	54 (67.5)	<0.001
No	26 (72.3)	0 (0.0)	26 (32.5)	
Total	36 (100.0)	44 (100.0)	80 (100.0)	
2) If the answer is yes, after that, do you wash your hands?				
Yes	2 (20.0)	26 (59.1)	28 (51.9)	0.026
No	8 (80.0)	18 (40.9)	26 (48.1)	
Total	10 (100.0)	44 (100.0)	54 (100.0)	
Knowledge				
3) The minimum time needed for handwashing after applying soap and water is:				
Less than 20 seconds	22 (61.1)	6 (13.6)	28 (35.0)	<0.001
More than 20 seconds	14 (38.9)	38 (86.4)	52 (65.0)	
Total	36 (100.0)	44 (100.0)	80 (100.0)	
Attitudes				
4) What practices should ideally be followed to prevent the spread of respiratory secretions?				
Coughing/sneezing on a disposable napkin/cloth handkerchief/hands and washing hands	10 (12.5)	41 (51.2)	51 (63.7)	<0.001
Keeping a distance from others while coughing/sneezing	2 (2.5)	12 (15.0)	14 (17.5)	
Covering the nose and mouth with hands and wiping hands on clothes after coughing/sneezing	14 (17.5)	3 (3.7)	17 (21.2)	
Do not know	12 (15.0)	2 (2.6)	14 (17.6)	
(* Multiple responses - total does not add to 100%)				
5) What practices should ideally be followed to dispose of the sputum of tuberculosis patients?				
Disposing of in the dustbin	7 (8.8)	13 (16.3)	20 (25.1)	<0.001
Disposing of in a container with lime and ash	0 (0.0)	8 (10.0)	8 (10.0)	
Pouring boiled water into the container	0 (0.0)	1 (1.3)	1 (1.3)	
Disposing of in a container with an antiseptic solution	0 (0.0)	3 (3.8)	3 (3.8)	
disposal in gutter/flushing in washbasin	16 (20.0)	28 (35.0)	44 (55.0)	
Dumping in the ground	0 (0.0)	1 (1.3)	1 (1.3)	
Do not know	17 (21.3)	3 (3.8)	20 (25.1)	

Knowledge of study participants towards causation and spread of PTB

Regarding the knowledge about TB causation, most of the study subjects responded that TB was a disease caused by germs (45, 56.25%), followed by the response “Do not know” (30, 37.5%), smoking (14, 17.5%), inadequate food intake (9, 11.25%), and worms and alcoholism (1, 1.25%), respectively. Regarding knowledge about the spread of TB, the majority (58, 72.4%) of the participants said that tuberculosis spreads through the inhalation route, followed by the response “Do not know” (17, 21.3%), ingestion route (5, 6.4%), and pricking and physical contact (1.3%, 1), respectively.

The majority (59, 74%) of the participants responded that the major source of PTB infections was respiratory secretions. But still, 17% (14) of the participants were not aware of the source of PTB infection. Around 9% (7) of the study participants said that the sources of PTB were blood, saliva, and faeces.

Only five out of 80 participants responded that they had enough knowledge about cough etiquette. Out of those five respondents, three of them said that they got that knowledge from healthcare professionals and the remaining two got it from mass media. Whereas, out of 80 participants, a good number (55%, 44) of participants responded that they had enough knowledge about hand hygiene. Out of those 44, the majority of them (59.1%, 26) said that they got that knowledge from mass media, followed by healthcare professionals (40.9%, 18). It was observed that nobody in our study responded as they had enough knowledge about ideal sputum disposal methods.

In our study, around 65% (52) of the study participants responded that the minimum time needed for hand washing after applying soap and water is more than 20 seconds. The majority (86.4%, 38) of the participants from the “above middle school” group and 38.9% (14) from the “up to middle school” group responded that the minimum time needed for hand washing after applying soap and water is more than 20 seconds. This difference was statistically significant (p = 0.000). However, the difference in responses on the duration of hand washing between the two socio-economic groups (“middle class and below” = 63.0% and “above middle class” = 69.2%) was not statistically significant (p = 0.582).

Attitude of study participants towards the safe sputum disposal methods

When the participants were asked about their attitude on safe & ideal sputum disposal methods, the majority of their responses were towards unsafe sputum disposal methods (81.3%, 65), whereas only 15% (12) of the responses were towards safe & ideal sputum disposal methods. Almost one-fourth (20, 25.1%) of the participants were in the view that they lack adequate information about safe & ideal sputum disposal methods.

Practices of study participants towards respiratory hygiene

Out of 10 presumptive PTB patients, five of them used to cover their nose and mouth with bare hands whereas the remaining five had the habit of covering with the clothes they wore while coughing and sneezing. Around 67.5% (54) of the participants responded that they practice covering their nose and mouth while coughing and sneezing, whereas 55% (44) of them have studied “above middle school”. Out of 54 participants who used to cover their nose and mouth while coughing and sneezing, 51.9% (28) responded that they used to wash their hands immediately. Of them, 26 studied “above middle school” and only two studied “up to middle school” (p = 0.026). Whereas, 20 of them belong to the above middle class and only eight of them belong to the “middle class and below” category of socio-economic status (p = 0.015). These differences were statistically significant, as given in Table 2.

**Table 2:** Knowledge and practices of respiratory hygiene and sputum disposal practices followed by the study participants according to socioeconomic class. * Multiple responses - total does not add to 100%. Fischer's exact test. A p-value of less than 0.05 is considered significant.

Knowledge and practices of respiratory hygiene & sputum disposal practices				
	Socio-economic status			
	Middle class and below	Above middle class	Total	P-value
	n (%)	n (%)	n (%)	
Practices				
1) Do you cover your nose and mouth while coughing/sneezing/blowing your nose?				
Yes	29 (53.7)	25 (96.2)	54 (67.5)	<0.001
No	25 (46.3)	1 (3.8)	26 (32.5)	
Total	54 (100.0)	26 (100.0)	80 (100.0)	
2) If the answer is yes, after that, do you wash your hands?				
Yes	8 (33.3)	20 (66.7)	28 (51.9)	0.015
No	16 (66.7)	10 (33.3)	26 (48.1)	
Total	24 (100.0)	30 (100.0)	54 (100.0)	
Knowledge				
3) The minimum time needed for handwashing after applying soap and water is:				
Less than 20 seconds	20 (37.0)	8 (30.8)	28 (35.0)	0.582
More than 20 seconds	34 (63.0)	18 (69.2)	52 (65.0)	
Total	54 (100.0)	26 (100.0)	80 (100.0)	
Attitudes				
4) What practices should ideally be followed to prevent the spread of respiratory secretions?				
Coughing/sneezing on a disposable napkin/cloth handkerchief/hands and washing hands	29 (36.2)	22 (27.5)	51 (63.7)	<0.001
Keeping a distance from others while coughing/sneezing	4 (5.0)	10 (12.5)	14 (17.5)	
Covering the nose and mouth with hands and wiping hands on clothes after coughing/sneezing	13 (16.2)	4 (5.0)	17 (21.2)	
Do not know	13 (16.3)	1 (1.3)	14 (17.6)	
(* Multiple responses - total does not add to 100%)				
5) What practices should ideally be followed to dispose of the sputum of tuberculosis patients?				
Disposing of in the dustbin	7 (8.8)	13 (16.3)	20 (25.1)	0.020
Disposing of in a container with lime and ash	1 (1.3)	7 (8.7)	8 (10.0)	
Pouring boiled water into the container	0 (0.0)	1 (1.3)	1 (1.3)	
Disposing of in a container with an antiseptic solution	1 (1.3)	2 (2.5)	3 (3.8)	
Disposal in gutter/flushing in the washbasin	31 (38.7)	13 (16.3)	44 (55.0)	
Dumping in the ground	1 (1.3)	0 (0.0)	1 (1.3)	
Do not know	17 (21.3)	3 (3.8)	20 (25.1)	

**Figure 1:**
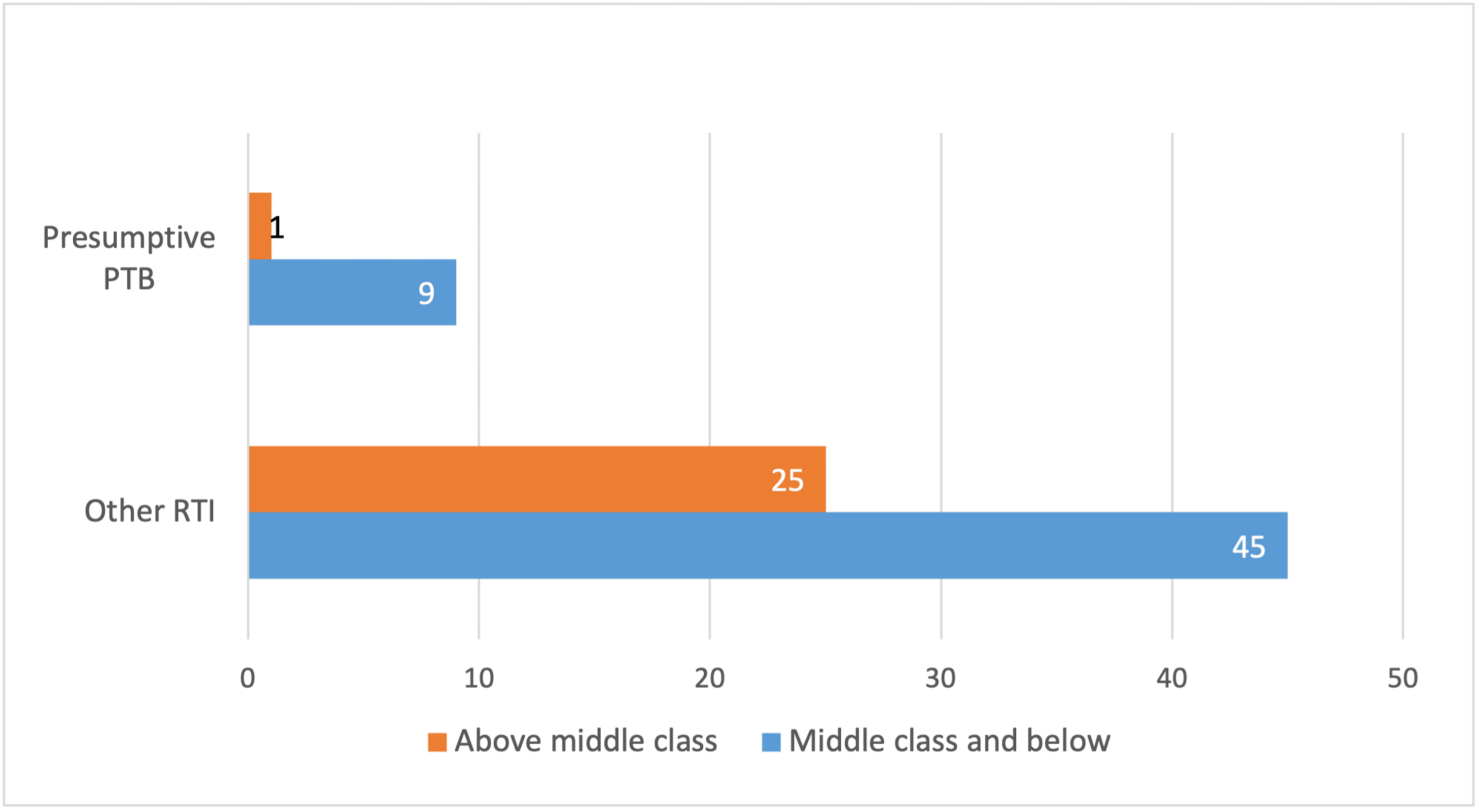
Presumptive PTB cases among different socio-economic groups. PTB: pulmonary tuberculosis; RTI: respiratory tract infection.

Figure 1 compares the number of presumptive PTB cases to other respiratory tract infections (RTIs) across two socio-economic groups: "middle class and below" and "above middle class". The figure shows that in the "middle class and below" group, out of 45 patients with RTIs, 20% (9) were presumptive PTB cases, representing a higher proportion of TB suspicion in this socio-economic category. In contrast, the "above middle class" group had 25 patients with RTIs, but only one (4%) was a presumptive PTB case. This stark difference highlights a socio-economic disparity, with presumptive PTB cases being more prevalent among individuals from lower socio-economic backgrounds.

## Discussion

Regarding the knowledge about TB causation, most of the study subjects responded that TB was a disease caused by germs (56.3%, 45). Also, the majority (72.4%, 58) of the subjects believed that TB spreads through the inhalation route. These findings are similar to the study by Bhattacharyya et al. [8]. Another study done by Sridevi et al. assessing the knowledge of TB spread revealed that 98% of the participants correctly responded that TB spreads through the airborne route since the study participants were healthcare workers in the study [9]. A study done by Dzeyie et al. revealed that the majority of the patients (63%) were aware that TB is a disease caused by germs, which is confirmatory with the present study finding despite the study participants were drug-resistant PTB cases in that study [10].

When the participants were asked about their attitude on safe & ideal sputum disposal methods, only 15% of the responses were towards safe & ideal sputum disposal methods. This is relatively low when compared with the findings on safe sputum disposal practices reported by Singh et al. and Rekha et al. (46.4% and 49.5%, respectively) [11,12]. This could be because those studies were done among sputum-positive PTB patients, who could have been trained for safe & ideal sputum disposal methods from their respective healthcare settings. Surprisingly, the finding is similar to another study done by Bhattacharyya et al. among sputum-positive PTB patients, where only 20.0% of the patients were following safe & ideal sputum disposal methods [8].

Seemingly, a good number of patients (67.5%) were covering their faces coughing and sneezing. This finding is similar to the studies by Bhattacharyya et al. (46.7%) and Barry et al. (69.1%) [8,13]. Additionally, the finding is better than the studies done by Bhat et al. and Nasreen et al., in which the practice was adopted by only 18.7% and 14.6% of the study participants, respectively [14,15]. Two different studies from Korea by Choi et al. and Kim et al. reported that the knowledge of cough etiquette was 56.1% and 54.9%, respectively [16,17]. These findings are similar to the present study findings. A study done by Alhazmi et al. among the Saudi Arabian general population found that 63.5% of them covered their noses and mouths with tissue paper when sneezing or coughing, which is almost similar to the current study finding [18]. More than half (51.9%) number of the patients wash their hands once they cover their nose and mouth with their hands while coughing and sneezing. The finding regarding whether the participants wash their hands after coughing/sneezing/blowing their nose tells that there are significant associations between the groups of both education as well as socio-economic classes. Therefore, higher education level as well as higher socioeconomic status positively affects the hand washing practice among study participants in the current study. It was understood that education level significantly (p = 0.000) affected the knowledge regarding the time required for hand washing among the study participants, whereas socio-economic status did not have a significant (p = 0.582) effect on the same. Therefore, higher education level positively affects the time required for hand washing among study participants in the present study. The India TB Report 2022 also reported that poverty and low socio-economic status predispose to detrimental outcomes like delayed care-seeking, increased default rates, and poor treatment outcomes [19]. The present study revealed a major deficit in practices despite having fair knowledge of respiratory hygiene and sputum disposal methods.

The study has some limitations as well. Since this study was done among patients with respiratory manifestations and presumptive pulmonary cases instead of PTB cases, the authors could not get many similar studies to compare the study findings. As we commonly see among hospital-based cross-sectional studies, the results obtained in this study are not generalizable to the general population since it is a hospital-based study. Recall bias can occur in such cross-sectional studies since the practices towards respiratory hygiene might vary each and every time when practising respiratory etiquette.

## Conclusions

This study highlights significant gaps in KAP regarding respiratory hygiene and sputum disposal methods among patients with respiratory manifestations in a primary healthcare setting. Despite moderate awareness about TB transmission routes, the majority of patients, especially those from lower socio-economic and educational backgrounds, lacked proper understanding and adherence to cough etiquette and safe sputum disposal practices. The study found a higher proportion of presumptive PTB cases among those in the "middle class and below" socio-economic group. This suggests a link between socioeconomic status and TB risk, reinforcing the need for targeted health education campaigns. Improved information, education, and communication (IEC) and behaviour change communication (BCC) interventions focusing on respiratory hygiene and TB prevention could significantly reduce the disease burden in communities at risk. Public health programs should prioritize educating lower socio-economic and less-educated groups to break the chain of TB transmission and improve overall respiratory health outcomes.
